# Zn/F-doped tin oxide nanoparticles synthesized by laser pyrolysis: structural and optical properties

**DOI:** 10.3762/bjnano.10.2

**Published:** 2019-01-02

**Authors:** Florian Dumitrache, Iuliana P Morjan, Elena Dutu, Ion Morjan, Claudiu Teodor Fleaca, Monica Scarisoreanu, Alina Ilie, Marius Dumitru, Cristian Mihailescu, Adriana Smarandache, Gabriel Prodan

**Affiliations:** 1 National Institute for Laser, Plasma and Radiation Physics (NILPRP), Atomistilor Str, no. 409, 077125, Bucharest-Magurele, Romania; 2 Ovidius University of Constanta, Mamaia Avenue no. 124, 900524, Constanta, Romania

**Keywords:** laser pyrolysis, nanoparticles, optical bandgap, Zn/F-doped SnO_2_

## Abstract

Zn/F co-doped SnO_2_ nanoparticles with a mean diameter of less than 15 nm and a narrow size distribution were synthesized by a one-step laser pyrolysis technique using a reactive mixture containing tetramethyltin (SnMe_4_) and diethylzinc (ZnEt_2_) vapors, diluted Ar, O_2_ and SF_6_. Their structural, morphological, optical and electrical properties are reported in this work. The X-ray diffraction (XRD) analysis shows that the nanoparticles possess a tetragonal SnO_2_ crystalline structure. The main diffraction patterns of stannous fluoride (SnF_2_) were also identified and a reduction in intensity with increasing Zn percentage was evidenced. For the elemental composition estimation, energy dispersion X-ray spectroscopy (EDX) and X-ray photoelectron spectroscopy (XPS) measurements were performed. In general, both analyses showed that the Zn percentage increases with increasing ZnEt_2_ flow, accompanied at the same time by a decrease in the amount of F in the nanopowders when the same SF_6_ flow was employed. The Raman spectra of the nanoparticles show the influence of both Zn and F content and crystallite size. The fluorine presence is due to the catalytic partial decomposition of the SF_6_ laser energy transfer agent. In direct correlation with the increase in the Zn doping level, the bandgap of co-doped nanoparticles shifts to lower energy (from 3.55 to 2.88 eV for the highest Zn dopant concentration).

## Introduction

Recently, there has been growing interest in the field of transparent conducting oxides and wide bandgap oxide nanocrystalline materials such as tin oxide (SnO_2_). It is generally agreed that SnO_2_ in its undoped form is an n-type semiconductor with a direct bandgap of 3.6 eV at room temperature. Its n-type conductivity is due to oxygen vacancies in its rutile structure. The bandgap, starting from the bulk value, increases as the size of the nanocrystal decreases, due to electron confinement at the nanoscale – the so called "quantum size effect".

Therefore, it is very important to synthesize nanoparticles with a narrow size distribution and with a desired mean diameter in order to control their optical and electrical properties [1]. The properties that make nanometer-sized SnO_2_ highly valuable from a technological point of view are its electrical conductivity, which is strongly affected by the surface states and the presence of dopants, transparency in the visible light range, high reflectance in the infrared range, and its classification as not potentially toxic or harmful [2–3]. The introduction of defects in the crystal lattice acts to gradually decrease the bandgap of SnO_2_, which extends the emission spectra to the visible light range, making these nanoparticles technologically very important for optoelectronic devices and photovoltaic systems. Theoretically, the reported value of the reduced bandgap of tin oxide nanoparticles by introduction of defects to the crystal lattice is ≈0.7 eV [4]. Generally, the doping of these semiconducting oxides with specific cations or anions is performed in order to increase their electrical conductivity while maintaining a high optical transparency in the visible range [5–6]. For the case of tin oxide, a comparative study using halogen anions (F, Cl, Br, I) as dopants shows that the fluorine anion induces the formation of thin films and exhibits the best performance in terms of transparency and conductivity [7]. Today, the most used material for commercial applications that require both transparency and electrical conductivity (employed in liquid crystal displays, organic light emitting diodes (OLEDs), touchscreens or in solar panels) is indium tin oxide (ITO), which unfortunately suffers from high cost and a limited supply of indium [5]. One promising, lower cost, but good performing material alternatives to ITO for these types of applications is fluorine-doped tin oxide (FTO) [8]. Regarding the cation doping for the synthesis of tin-based transparent and conductive oxidic (TCO) materials, the literature has been focused on doping with i.) antimony by spray pyrolysis [9] or by sol–gel methods followed by spin-coating and annealing in different environments [10], ii.) manganese by long-time annealing of Mn/SnO_2_ bilayers in air at 200 °C [11] or by co-precipitation [12], iii.) aluminum, copper or indium all by spray pyrolysis from ethanolic solutions [13] and iv.) iron by laser pyrolysis [14–15] or by electron beam evaporation [16]. Cobalt-doped tin oxide has also been reported, and the resulting polycrystalline films were prepared by spin-coating and annealing from chloride ethanolic solutions resulted in lower bandgap values than pure SnO_2_ [17]. Highly conductive films based on amorphous Co-doped SnO_2_ were also synthesized using a pulsed spray evaporation chemical vapor deposition (CVD) technique [18]. One of the most reported cationic dopants for tin oxide is Zn^2+^, where the obtained zinc-doped tin oxide (ZTO) films show lower bandgap values (3.6–3.7 eV for 5–10 wt % Zn) than the undoped ones (3.97 eV). All of these were synthesized by sol–gel methods from SnCl_2_ and ZnCl_2_ hydropropanolic solutions and short-time annealed at 480 °C [19]. The same tendency was found in the sprayed (from SnCl_2_ and Zn(CH_3_COO)_2_ in acidic H_2_O–CH_3_OH solutions) and 400 °C heated films where the bandgap decreased from 3.85 eV down to 3.57 eV with increasing Zn weight concentration in the initial solution from 0 to 25% [20]. The facile substitution of Zn^2+^ into the SnO_2_ lattice containing Sn^4+^ cations can be explained by the similar values of their ionic radii (0.74 Å for Zn^2+^ and 0.71 Å for Sn^4+^ [21]) and thus each zinc ion replaces a tin ion accompanied by the appearance of an oxygen vacancy (equivalent with two holes) to maintain the crystal electrical neutrality. Moreover, it was hypothesized that part of the zinc ions can also occupy the interstitial sites in the SnO_2_ lattice, as reported for the Zn-doped SnO_2_ nanoparticles obtained by the microwave solvothermal process, where a highly nonlinear relation was found between the zinc atomic doping percent and bandgap values [21]. Another strategy for improving the properties of tin oxide consists of the simultaneous doping with both cations and anions. In the case of a high aluminum and lower sulfur co-doped film made by spray pyrolysis at 480 °C, the transparency increased and the grain size was significantly reduced for the optimal composition [22]. This approach benefits from the reduction of the number of recombination centers and enhancement of “the electron–hole pair separation to stimulate the change in the bandgap by eliminating the impurity states” by charge compensation between positive and negative ion dopants, and it can also “facilitate the overall mixing of the impurity states and VB/CB” (valence band/conduction band) “by adjusting the position of bandgap to obtain an optimized narrow value” [23]. Thus, using a solution-based single-source precursor (Er-doped KSnF_3_), oxygen-vacancy-rich nanocrystals of co-doped Er and F SnO_2_ were obtained at low temperature with an estimated 4.18 eV bandgap value [24]. Also, the 1 atom % Nd-doped FTO film obtained by spray pyrolysis at 500 °C presented the lowest sheet resistance and resistivity values, which was accompanied by a 4.15 eV bandgap – a value 4.21 eV lower than that of FTO obtained under similar conditions. Also, in this case, the bandgap values decreased with increasing metal dopant content, down to 3.93 eV for 4 atom % Nd [24].

The FTO films were also successfully tested for other applications such as anticorrosive coatings on steel for fuel cell bipolar plates [25], sensors for liquefied petroleum gas [26], photocatalysts for rhodamine 6G dye degradation in aqueous solution [27] and were proposed as a thermal UV sensor for high-radiation environments [4]. Moreover, the ZTO materials were also employed as volatile organic compound (VOC) (such as methanol, ethanol or acetone vapors) sensors [28], as an anode for Li-ion microbatteries [29], as photocatalysts for brilliant green dye degradation in solution under solar light [30] and even as a component for supercapacitors [31].

The in situ synthesis of SnO_2_-based nanoparticles co-doped with F and Zn is demonstrated in this work. For this purpose we use the pyrolysis of SnMe_4_ and ZnEt_2_ sensitized with a SF_6_ gas flow, all in oxidative mixtures. We also report a study on the structural, optical and electrical properties of such Zn/F co-doped SnO_2_ nanoparticles. Depending on the experimental parameters, different Zn and F doping levels in SnO_2_-based nanocrystals were obtained. To the best of our knowledge, Zn/F-doped tin oxide nanoparticles with low Zn and high F content have been prepared for the first time. Related fluorine-doped zinc tin oxide (FZTO) thin films with higher zinc concentration (from 5.5 to 35.5 atom %) and lower fluorine content (0.62 to 3.49 atom %) made by spray pyrolysis showing high transparency and bandgap values between 3.86 and 4.45 eV have also been reported [32]. Other researchers have used radio frequency magnetron sputtering of mixed 30 wt % ZnO and 70 wt % SnO_2_ targets to obtain similar FZTO films, yet their reported different bandgap values were shown to increase with the vacuum annealing treatment temperature (from 3.41 eV at 300 °C to 3.60 eV at 600 °C), where the amorphous to crystalline conversion was observed only at 600 °C, accompanied by an almost complete fluorine loss [33].

## Results and Discussion

### Structural properties

**Figure 1:**
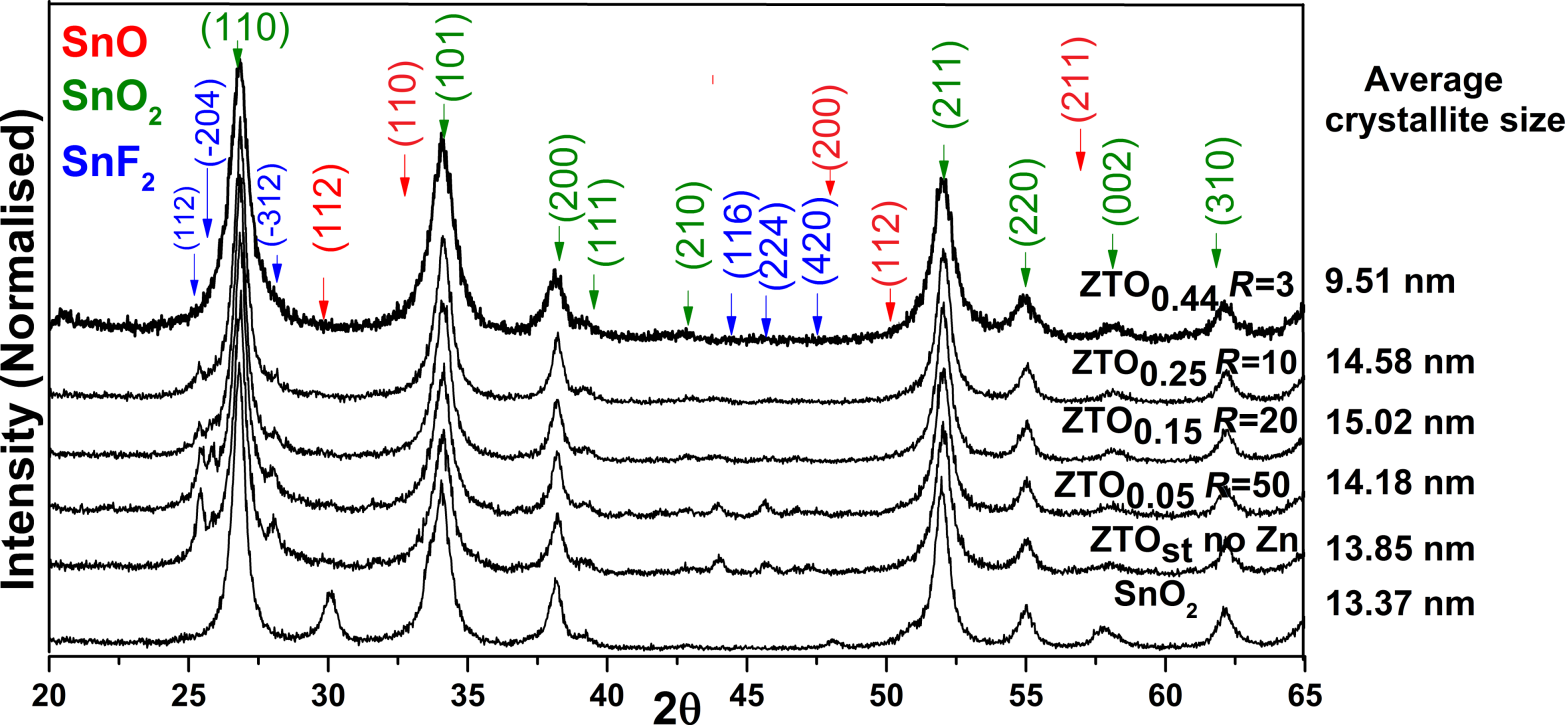
XRD spectra of the Zn/F co-doped, F-doped and undoped SnO_2_ nanoparticles.

The X-ray diffraction (XRD) patterns of Zn/F co-doped, F-doped and undoped SnO_2_ nanoparticles are superposed in Figure 1. The ratio, *R*, between the SnMe_4_ and ZnEt_2_ flows is shown on the right of the figure, near the sample name. In all cases, each XRD pattern clearly demonstrated the nanocrystalline feature of the analyzed powders. The most evident diffraction peaks (see the green arrows) correspond to the tetragonal rutile structure of the SnO_2_ phase (PDF No: 00-041-1445); thus the identified peaks centered at 26.8°, 34°, 38°, 51.9°, and 54.8° can be assigned to (110), (101), (200), (211) and (220) crystal planes of this phase, respectively. Also the main diffraction patterns of stannous fluoride, SnF_2_ (PDF No: 00-015-0744 blue arrows), and stannous oxide, SnO (PDF No: 04-005-4541 red arrows), are distinguished in those samples with a low Zn doping level (ZTO_0.05_, ZTO_0.15_ and ZTO_0.25_) as well as in the only F-doped (ZTO_st_) and undoped (SnO_2_) powders. The most significant peaks of the SnF_2_ phase are identified for the ZTO_st_ powder, where their intensity tends to decrease with increasing Zn percentage. No phases corresponding to zinc or other zinc compounds are observed in any of the analyzed samples. For instance, regardless of the Zn doping degree, the presence of the main ZnO peak (101) at 2θ ≈ 36.5° was not distinguished. This result highlighted that at this Zn doping level there is no segregation process toward some Zn-rich crystalline phases such as Zn_2_SnO_4_. It is worth to noting that the undoped reference sample is the only one where the clear signature of a stannous oxide phase can be observed, even when the amount of tin precursor and oxygen used for their synthesis is the same as that used for the ZTO_st_ sample, where SF_6_ was used as a sensitizer instead C_2_H_4_. Due to the better infrared absorption coefficient of SF_6_, we had to employ a larger amount of C_2_H_4_ to obtain the same laser-induced heating effect. Thus, the C_2_H_4_ competes with the Sn(CH_3_)_4_ vapors for the limited quantity of available oxidizing agent (O_2_), resulting in a mixture of SnO_2_ and SnO phases in the case of the undoped sample. We previously reported a similar oxygen deficient environment using tetramethyl tin laser pyrolytic oxidation, where metallic tin was also found in addition to SnO_2_ and SnO phases [14].

The presence of fluorine is explained by the catalytic decomposition of the sensitizer that acts also as a fluorine donor. A probable explanation of the SnF_2_ XRD pattern intensity decrease with increasing Zn doping is the presence of ZnEt_2_ that acts as an inhibitor of SF_6_ decomposition, in particular, by lowering the temperature in the synthesis zone (it decreases gradually from 615 °C at the ZTO_st_ sample to 560 °C for the ZTO_0.44_ sample). These observations lead to the conclusion that the dominant crystalline structure is SnO_2_, with the secondary phase of SnF_2_ in the case of small Zn doping levels, and the Zn atoms probably substitute Sn sites in the oxide phase, thus changing the optical and electrical properties of the ZTO_st_ standard sample. Yet, the simultaneous substitutional fluorine doping of SnO_2_ must also be considered, as will be discussed in the paragraph when XRD and XPS analyses are presented. Considering the most intense peak (101) of the dominant phase SnO_2_, the crystallite size (*D*) was calculated using Scherrer's equation:


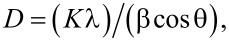


where the dimensionless shape factor *K* is 0.9, the X-ray wavelength λ is 1.5418 Å, while β is the peak full-width at half-maximum (FWHM) and θ is the Bragg angle. The FWHM was evaluated for the first three peak profiles ((110), (101) and (200)) using a pseudo-Voigt function. In all cases, except the ZTO_0.44_ sample, the crystalline size remains constant around 14 nm, but for the highest Zn doping level, a significant crystalline size decrease was observed. This tendency is correlated with the flame temperature decrease generated by decreasing *R* = *D*_SnMe4_/*D*_ZnEt2_, while keeping the sensitizer flow constant.

In order to estimate the elemental composition for the Zn/F-doped SnO_2_ powders, EDX and XPS measurements were conducted; the results are presented in Table 1. In the XPS measurements, the peaks centered around 487 eV, 494 eV, 531.43 eV, 684 eV, 1022 eV and 1045 eV were assigned to Sn3d_5/2_, Sn3d_3/2_, O1s, F1s, Zn2p_3/2_ and Zn2p_1/2_, respectively, based on earlier reports [23,34]. In general, both analyses are in agreement with the composition of the reactive gas mixture. Thus, the zinc percentage in powder increases with increasing ZnEt_2_ flow. The presence of carbon in the powder is minor and is derived from the decomposition of methyl and ethyl radicals released by volatile organometallic compounds. This fact can be explained by an insufficient (less than stoichiometricly required) quantity of oxidant for the total oxidation of alkylmetallic precursors to oxides (SnO_2_ and ZnO, in fact Zn-doped SnO_2_), CO_2_ and H_2_O in the laser pyrolysis reaction zone. Due to the much higher reactivity of metals, the metal oxides are formed with priority, and the unoxidized alkyl radicals from the precursors will undergo a complex process involving reactions such as dimerization, dehydrogenation, polymerization, reticulation, aromatization and carbonization with the final formation of amorphous hydrogenated carbon. For the ZTO_0.44_ case, in spite of an apparent oxygen excess, the carbon is still formed (the EDX-extracted carbon atomic percent is the smallest from all ZTO powders) and this fact can be attributed to an insufficient diffusion between the oxygen from the annular flow and alkylmetals vapors from the central flow. Also, for the undoped sample, the ethylene sensitizer seems to be another major carbon source in the resulting metal-oxide-based powder, similar to the process of ethylene-sensitized TiCl_4_ oxidative laser pyrolysis that we previously reported [35–36].

**Table 1:** Evaluation of the elemental composition by EDX and XPS analyses.

Sample	Elemental composition (atom %)									
	C		O		Sn		F		Zn	

	EDX	XPS	EDX	XPS	EDX	XPS	EDX	XPS	EDX	XPS
ZTO_st_	9.29	6.97	45.37	38	32.47	21.6	12.87	27	0	0
ZTO_0.05_	9.18	7.3	49.16	42	31.91	26	9.75	21.6	Tr.^a^	0
ZTO_0.15_	8.67	6.57	56.15	50.53	30.68	25.29	4.38	16.69	0.12	0.25
ZTO_0.25_	9.09	9.40	54.26	48.16	30.92	22.24	5.45	15.30	0.29	0.25
ZTO_0.44_	8.18	14.96	54.22	53.08	27.22	15.02	6.64	12.39	3.74	4.55

^a^Tr. means less than 0.1 atom % Zn.

Also, the elemental estimations revealed the significant presence of F in all as-synthesized powders in accordance with the SnF_2_ phase identified in XRD patterns. As discussed before, the fluorine presence in nanoparticles may be explained by the catalytic partial decomposition of SF_6_. Typically, this gas is stable in the temperature range chosen for these experiments: 500–650 °C. An increased concentration of F is observed for XPS analysis; this is explained by a compositional gradient in the nanopowder (the superficial F concentration is higher than in the inner zone of the nanoparticle).

The XPS analysis is only sensitive to surface areas, with a penetration depth of a few nanometers, while EDX evaluates the average composition on the irradiated area (containing more than 1000 particles in average), thus its penetration depth exceeds the mean particle size (few tens of nanometers). Consequently, the predominant presence of F at the nanopowder surface may be evidence for the assumption that freshly formed nanoclusters have a catalytic role in the partial decomposition of SF_6_.

The elemental analysis reveals that the laser pyrolysis method can be used to synthesize nanoparticles with a large variation of Zn doping (from 4 to 0.1 atom %), by controlling only the SnMe_4_ to ZnEt_2_ ratio in the reagent mixture. Also, there is no clear evidence of sulfur in any of the synthesized powders either from EDX analysis (no peaks around 2.13 KeV – S Kα emission line) or XPS spectra (no peaks or shoulder around 164 eV ascribed to S2p_3/2_ binding energy). This fact may be explained by the following decomposition reaction: SF_6_ → SF_4_ + F_2_ [37] that seems to be catalyzed in our case by the freshly formed tin-based clusters. Using the same SF_6_ flow rate (0.5 sccm), the highest F content was found in the ZTO_st_ sample where none of the central Ar streams pass through ZnEt_2_. The most probable explanation comes from the reactive flame temperature when the presence of ZnEt_2_ vapors slightly cools the flame due to its endothermal decomposition. This consequently creates a hotter reaction zone – and as in the case of the ZTO_st_ sample synthesis without the zinc precursor, this favors the SF_6_ decomposition with fluorine release. The presence of an increased Zn content is observed in the ZTO_0.44_ sample and is directly dependent on the Zn/Sn vapor flow ratio.

**Figure 2:**
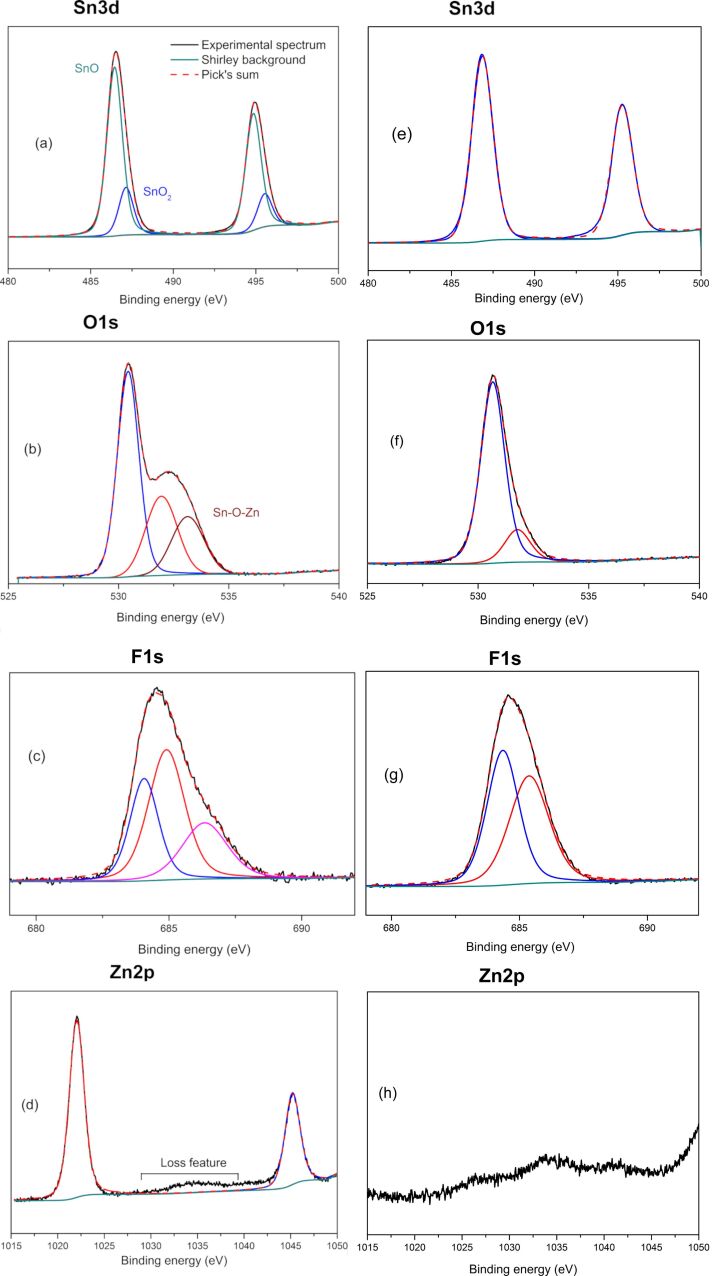
XPS high-resolution spectra of: (a, b, c, d) Zn/F-doped SnO_2_ nanoparticles (sample ZTO_0.44_) and (e, f, g, h) F-doped SnO_2_ nanoparticles (sample ZTO_st_).

High-resolution XPS core level spectra of Sn3d, O1s, F1s and Zn2p were made for the highest Zn-doped sample (ZTO_0.44_) and the only the fluorine-doped sample (ZTO_st_). The binding energies were calibrated using the C1s peak at 284.4 eV in order to compensate the surface charging effects. From Figure 2 it can observed that in the case of ZTO_st_ sample, Sn exhibits only one oxidation state, while the Zn doping is accompanied by the formation of a secondary SnO phase, see Figure 2a. Also, the binding energy of Sn in the Zn-doped SnO_2_ nanoparticles slightly decreases with the Zn doping degree from 486.8 to 485.15 eV. This behavior was previously observed in the hydrothermally synthesized Zn-doped powder and it can be attributed to: (i) changing effects, or, (ii) to the oxygen deficiency since the Sn binding energy changes with Sn oxidation nature [34]. Furthermore, a significant contribution may come from the presence of Sn–F bonds because they have a higher Sn3d_5/2_ binding energy: 487.2 eV for SnF_2_ [38].

Figure 2b and 2f present a comparison of the O1s transition peak. For the ZTO_st_ sample, the observed O1s binding energy has a major peak centered at 530.65 eV, which corresponds exactly to the value for SnO_2_. The second peak centered at 531.8 eV can be attributed to the O–C bond [39] or to O^2−^ in the oxygen deficient regions [40]. In the case of sample ZTO_0.44_ the main O1s peak shifts to a lower value: 530.45 eV; we observed that this trend is correlated with the Zn doping degree. The third peak could be assigned to Sn–O–Zn coordination [41]. As shown in Figure 2d, the binding energy of the Zn2p_3/2_ and Zn2p_1/2_ transition peaks is 1021.7 and 1044.9 eV, respectively, which confirm the presence of Zn in the doped SnO_2_ nanoparticles, and possible traces of Zn in the only fluorine-doped sample, probably due to powder collector contamination from previous experiments. The F1s spectrum (Figure 2c) consists of three peaks originating from: ZnF_2_ at 684.1 eV, SnF_2_ at 684.9 and partially fluorinated carbons (contamination from the interaction of fluorine provided by SF6 decomposition with the alkyl radicals from metallic precursors) at 686.35 eV. The ≈685 eV F1s XPS peak was also detected in fluoride-doped SnO_2_ from milled PVdF (polyvynilidene fluoride)/SnO_2_ mixtures [42] and in solid or hollow fluoride-mediated hydrothermal synthesized SnO_2_ nanostructured microspheres [43]. Moreover, for the half hour milled sample from [42], the stannous fluoride XRD peaks can also be identified at 2θ ≈ 25° and 2θ ≈ 27°. In brief, the XPS results indicate that Zn atoms are incorporated into the SnO_2_ crystal lattice through the substitution of Sn sites.

**Figure 3:**
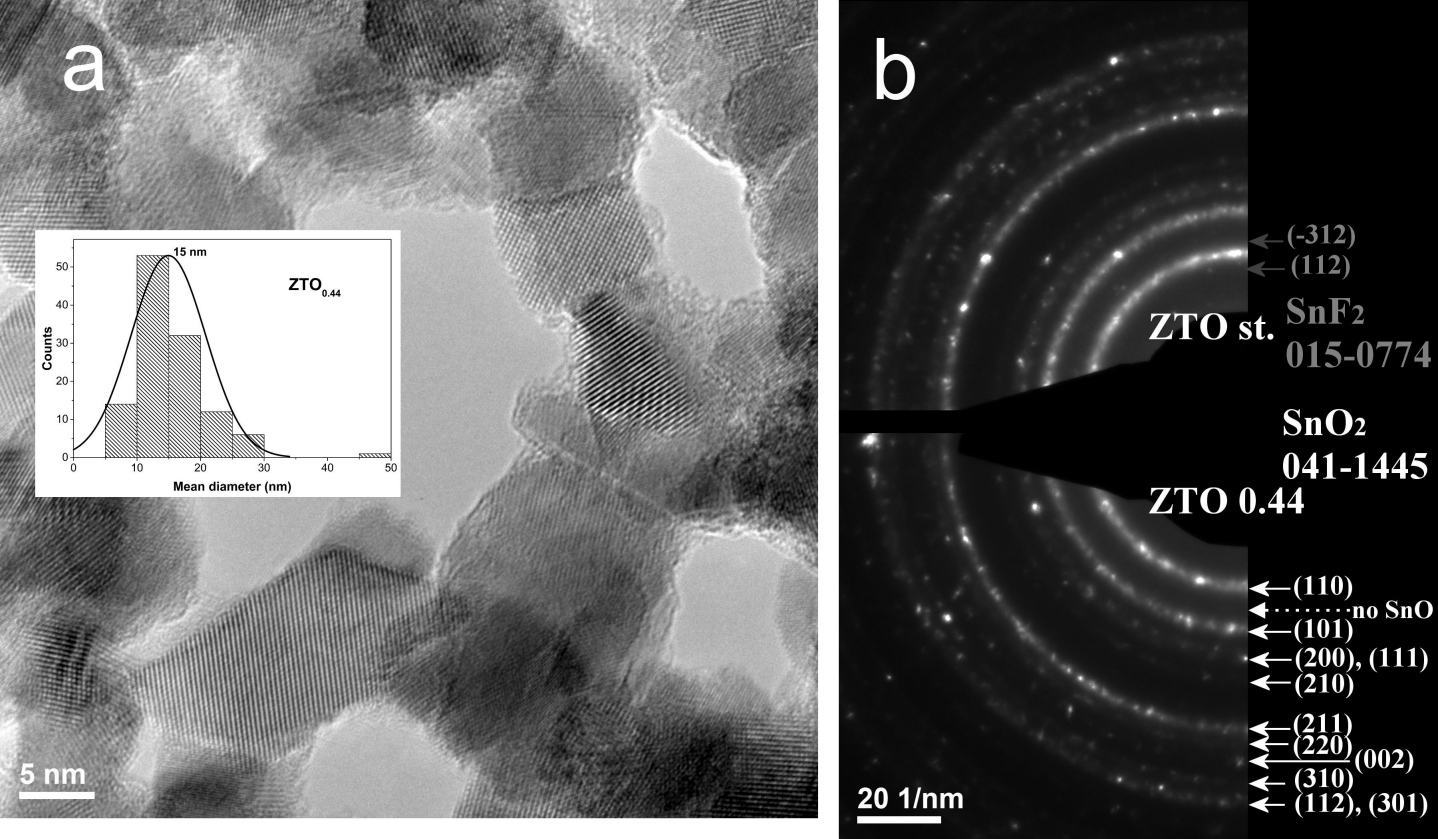
a) HRTEM image of sample ZTO_0.44_ and its mean size distribution; b) SAED patterns of ZTO_st_ (top) and ZTO_0.44_ (bottom) samples.

In Figure 3a, a high-resolution TEM (HRTEM) image of a Zn/F-doped SnO_2_ sample (labeled ZTO_0.44_) and its mean size distribution (inset in Figure 3a) are presented. The polyhedral crystalline tin dioxide aggregated nanoparticles can be clearly seen in the HRTEM image. Also, a very thin disordered layer can be identified on the crystallite surface, most likely being composed of the amorphous (hydrogenated) carbon derived from alkylmetal precursors as discussed before.

The values of the mean diameters for all the doped samples are shown in Table 2. It can be seen that in the ZTO_st_ and ZTO_0.05_ nanopowders, by increasing the degree of F doping, and in the (quasi)absence of Zn, the crystallite size and the average particle diameter decreases. One also can notice that the smallest mean crystallite size (9.51 nm) can be found for ZTO_0.44_ powder, where the F doping level is the lowest (yet not zero) while those of Zn is the highest. Also, when the F and Zn doping concentration is reduced (from ZTO_0.44_ to ZTO_0.25_ and to ZTO_0.15_), an inverse tendency of increasing crystallite size was observed (from 9.51, 14.58 and 15.02 nm, respectively), yet without a clear correlation with the average diameter of the nanoparticles measured from TEM images. The tendency of the reduction in the mean crystallite size with increasing Zn concentration (without ZnO phase segregation) was also observed for the case of Zn-doped SnO_2_ nanocrystals prepared by a solid-state reaction from stannous chloride and zinc acetate, yet in this case, a mixture of tetragonal rutile-type and minority orthorhombic tin dioxide phases was identified [44].

**Table 2:** Dependence of the mean diameter and crystallite size with F and Zn (atom %) doping level.

Sample	Mean diameter (nm)	Mean XRD crystallite size (nm)	EDX		
			F (atom %)	Zn (atom %)	Total F + Zn (atom %)

ZTO_0.05_	18	14.18	9.75	0	9.75
ZTO_st_	14	13.85	12.87	0	12.87
ZTO_0.44_	15	9.51	6.64	3.74	10.38
ZTO_0.25_	18.5	14.58	5.45	0.29	5.74
ZTO_0.15_	15	15.02	4.38	0.12	4.5

The crystalline phases identified from SAED (see Figure 3b) images are consistent with those from XRD, indicating structural uniformity up to the level of nanoparticle agglomeration containing several dozen crystalline domains. From the superposed SAED images of the ZTO_st_ sample (Zn-free, only fluorine doped) (top) and the ZTO_0.44_ sample (bottom), it can be observed that almost all diffraction rings belong to the SnO_2_ phase (PDF No: 00-041-1445). No rings ascribable to the maximum diffraction planes of SnO (110) and SnF_2_ (112) as secondary phases can be identified in the SAED image of Zn-doped sample (ZTO_0.44_). For the ZTO_st_ sample, the presence of a few SnF_2_ crystals is probable. In the SAED image of this fluorine only doped tin oxide powder, we have identified diffraction dots positioned towards the center in agreement with the most intense crystalline plane planes (112) and (−312) of the SnF_2_ phase. Also, in both SAED images there are no diffraction rings or dots placed in ZnO(101): 2.48 Å and Zn(101): 2.09 Å positions.

### Raman spectroscopy

**Figure 4:**
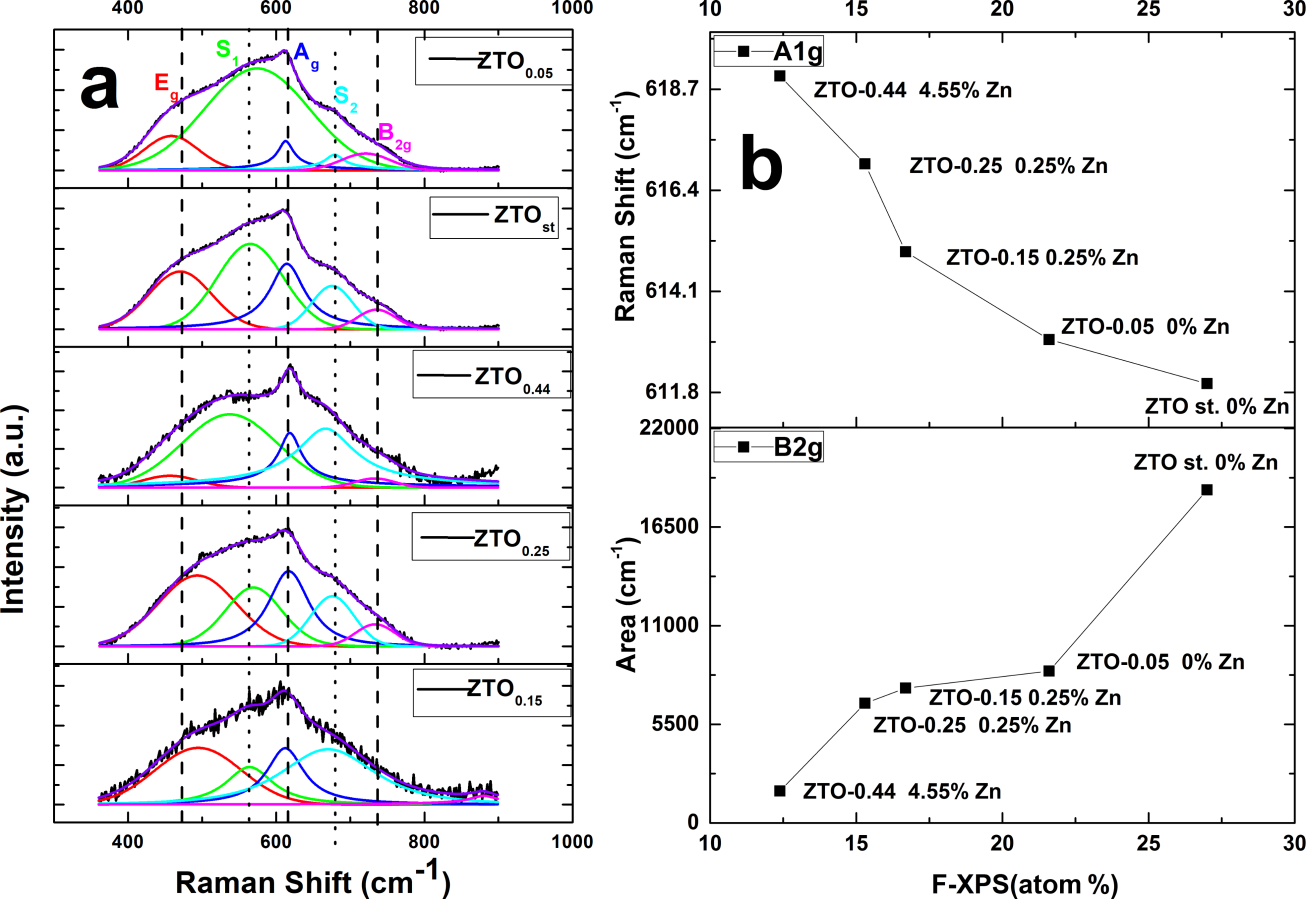
a) The Raman spectra of the as-prepared samples; b) The dependence from the Raman and XPS analysis.

Raman spectroscopy, commonly employed to provide qualitative information via phononic behavior regarding the crystalline nature of materials, is a useful tool for investigating disorder in oxide materials. Figure 4a presents a typical room temperature Raman spectra of as-synthesized F or Zn/F-doped SnO_2_ nanopowders. The Raman spectra analyzed in this work does not include the 1300–1500 cm^−1^ window. The carbon Raman component has not been approached in this analysis due to low amorphous C content in the samples, which, combined with their photoluminescent feature, makes the Raman C vibrational modes too weak to be clearly distinguished in the measured spectra against the background noise.

Three fundamental Raman peaks are observed at around 470, 618 and 730 cm^−1^, corresponding to E_g_, A_1g_, B_2g_ first-order Raman active modes of rutile-type phase of SnO_2_ [45–47]. The B_2g_ and A_1g_ modes are both related to the contraction and expansion of Sn–O bonds in the perpendicular plane to the *c*-axis. The E_g_ mode is related to the oxygen vibration in the direction of the *c*-axis [48–49]. According to Diéguez et al. [49], the band S1 located at around 570 cm^−1^ is linked to amorphous SnO_2_. The correlation between the intensity of the Raman band with the size of SnO_2_ nanoparticles is also revealed.

For samples presented in this article, the double doping with F/Zn of SnO_2_ nanoparticles leads to different dependencies. In Figure 4a the Raman spectra and the corresponding bands after their deconvolution with a Pearson 7 function are presented. After the deconvolution, a correlation can be made between the fluorine doping atomic percent (from the XPS analysis) and the Raman shift value of the A_1g_ band or the B_2g_ peak area as shown in Figure 4b. Thus, with the increase of fluorine content in the powders, the A_1g_ position shifts toward lower wave numbers, whereas the B_2g_ band area increases about 100 times.

The F and Zn content, in infrared-laser-assisted synthesized SnO_2_ nanoparticles, shifts the A_1g_ Raman mode also due to a cumulative effect of an increase in the crystallite size combined with Zn doping of rutile crystalline structure. One can observe (Figure 4b) a direct dependence of the mode frequency with XPS Zn content detected on the nanoparticle surface. This shifting correlated with the B_2g_ Raman mode area increase indicates nanoparticle surface disorder induced by F attachment.

S1 and S2 bands appear as a disorder activation consequence [25] inferred by F, Zn and nanocrystallite size. When the F concentration is high (without Zn doping) the disorder is considered to be surface induced, while for a sample with a high Zn concentration (EDX – 3.74 atom %), ZTO_0.44_, the disorder is volume generated, along with a depreciation in the crystalline quality with respect to SnO_2_ size. This behavior is sustained by the E_g_ mode extinction in the last sample.

### Optical and electrical properties

**Figure 5:**
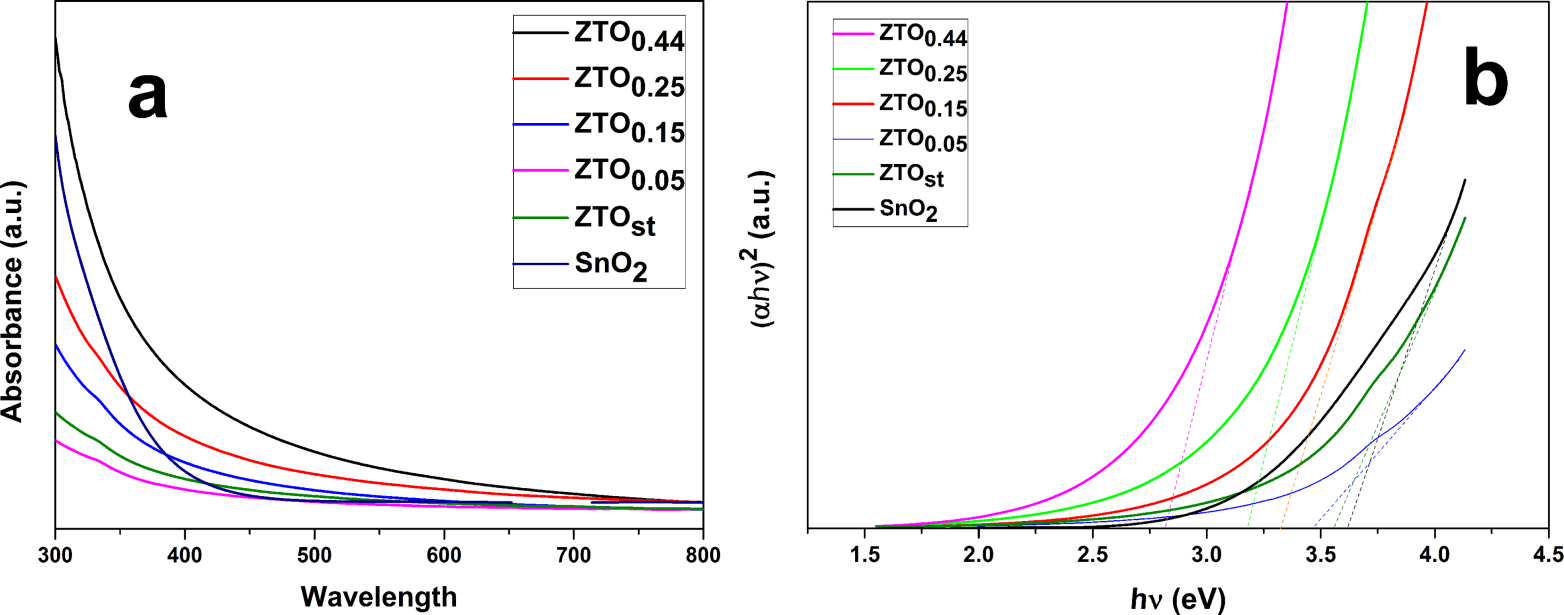
a) Optical absorption spectra of the nanoparticles, b) Tauc’s plot for Zn/F-doped and undoped SnO_2_ nanoparticles (α*h*ν)^2^ versus (*h*ν).

Figure 5a shows UV–vis absorbance spectra of only F-doped and Zn/F co-doped SnO_2_ nanoparticles. It is well known that the absorbance depends on factors such as the bandgap, grain size, oxygen deficiency, surface roughness, and impurity centers [21].

The direct bandgaps of the nanoparticles were determined from the Tauc relation [50–52] given by:


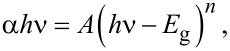


where α is the absorption coefficient, *A* is a constant, *h*ν is the photon energy, *n* is an index that can take different values depending on the type of transition. In this case *n* equals to ½, corresponding to a direct transition. The optical energy gap *E*_g_ can be estimated by plotting (α*h*ν)^2^ versus (*h*ν), then by extrapolating the linear portion of the absorption edge to the photon energy axis at the value (α*h*ν)^2^ = 0.

As shown in Figure 5b and reported in Table 3, the bandgap energy of the SnO_2_ materials show a decrease with decreasing F doping concentration and with the increase of Zn doping concentration. The bandgap of our standard undoped sample was found to be 3.55 eV, which is lower than that of bulk SnO_2_ (3.6 eV). This may be attributed to the impurity clustering which occurs in heavily doped semiconductors. Also, the carbon presence in all (including the reference without F and Zn) of our laser-synthesized tin-oxide-based nanopowders can also influence the optical properties, including the bandgap values. A clear optical behavior influence of the presence of carbon layers on tin dioxide can be observed for the SnO_2_@C and SnO_2_@SiO_2_@C nanostructured microspheres (C symbolizing here reduced graphene oxide, rGO) reported in [53], where the UV–vis spectra show a clear increase in absorbance (mostly in the visible domain, but also in the 320–400 nm UV zone) when compared with pure SnO_2_. Consequently, the resulting bandgap values diminished from 3.6 eV in pure SnO_2_ down to 3.2 eV for both SnO_2_@SiO_2_@C and SnO_2_@C samples [53]. A similar behavior can also be supposed for our carbon-containing tin oxide samples, yet in our case, the UV–vis absorption in this region seems to be considerably lower. Unfortunately, in the methods employed to extract the absorbance (diffuse reflectance spectroscopy using BaSO_4_ reflectance standard in [53] vs direct absorption from ethanolic suspensions in our study), the different carbonaceous structure of the coating and the use of arbitrary units make a direct comparison difficult. Regarding the zinc ion doping bandgap influence for the Zn/F co-doped SnO_2_ systems, a previous report showed a similar tendency with ours of decreasing bandgap (from 4.40 to 4.13 and to 3.86 eV) with increasing Zn content (from 0.5 to 1.5 to 2.3 surface atom % Zn doping) for low F doping levels (0.37, 0.53 and 0.29 atom % F, respectively) and heavy carbon surface contaminated tin dioxide films [32]. The authors discussed two possible reasons for this decreasing trend that also can be considered for our powders: the lower bandgap value for ZnO (3.2 eV) vs that of SnO_2_ (3.8 eV) and the "formation of Urbach tails at high impurity concentration" [32]. Moreover, as highlighted in the introduction, some sprayed Zn-doped SnO_2_ films also show the same tendency [20]. The energy bandgap measurements can help in engineering so that Zn/F-doped SnO_2_ can be considered as a typical transparent conducting oxide (TCO) suitable for solar cell applications.

**Table 3:** Dependence of the bandgap and resistivity (ρ) with F and Zn (atom %) doping level.

Sample	Bandgap (eV)	F atom %	Zn atom %	Ρ (Ω·cm)

ZTO_st_	3.55	27	0	156.18
ZTO_0.05_	3.47	21.6	0	149.33
ZTO_0.15_	3.3	16.69	0.25	65.76
ZTO_0.25_	3.18	15.30	0.25	57.54
ZTO_0.44_	2.83	12.39	4.55	1246.7

**Figure 6:**
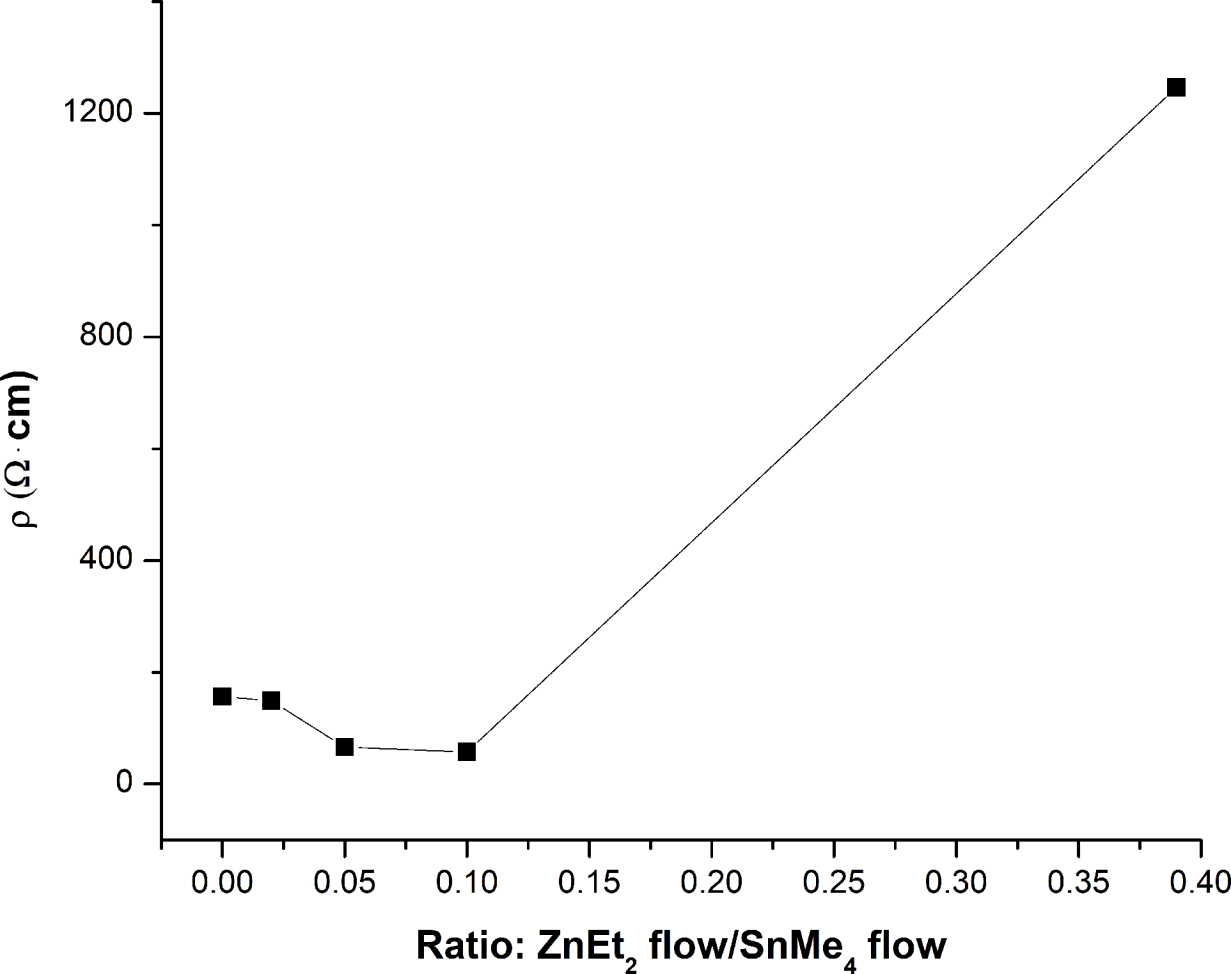
Electrical resistivity measurements.

Thin films were prepared from the as-synthesized powders using spin-coating and found to have an apparent density of around 0.8 g/cm^3^. The resistivity at room temperature is on the order of tens to hundreds of Ω·cm. These electrical values are in agreement with those reported in literature considering that the resulting films have a low particle packing density (SnO_2_ bulk density is 7.0 g/cm^3^). Zn-doped SnO_2_ films have resistivity values slightly lower than the only F-doped ones (Figure 6 and Table 3, last column), and have an optimum Zn doping level for sample ZTO_0.25_ (ZnEt_2_ flow/SnMe_4_ flow = 0.1).

## Conclusion

The laser pyrolysis technique has been successfully employed to obtain Zn and F co-doped SnO_2_ nanopowders, according to XPS and EDX analyses. The dominant crystalline structure, as indicated by XRD, is SnO_2_ with the secondary phase of SnF_2_ in the case of small Zn and high F doping levels. The mean crystallite size is ≈14–15 nm, but at the highest Zn doping level, a significant crystalline size decrease down to ≈9 nm was observed. The crystalline phases identified from SAED images are consistent with those resulting from XRD, indicating that almost all diffraction rings belong to the SnO_2_ phase. Three fundamental Raman peaks are observed at around 470, 618 and 730 cm^−1^, corresponding to first-order Raman active modes, E_g_, A_1g_, B_2g_ of rutile phase of SnO_2_. It has been demonstrated that by the increasing the fluorine concentration, the A_1g_ position shift toward lower wavenumbers and the B_2g_ band area grows about 100 times. The optimum resistivity of the co-doped SnO_2_ films is 57.54 Ω·cm for a ZnEt_2_ to SnMe_4_ flow ratio of 0.1. In order to understand the effect of zinc and fluorine addition on the optical and electrical properties for TCO applications, further experiments are planned and results will be reported in the future.

## Experimental

Laser pyrolysis is a versatile method to synthesize nanoparticles. The process is based on the resonance between the emission line of a continuous wave CO_2_ laser (λ = 10.6 μm) and the infrared absorption band of at least one gas-phase component. An additional substance, the so-called sensitizer, is used in the case of non-absorbing gas/vapor precursors. The experimental arrangement consists of a heat-resistant glass reactor with a cross-sectional configuration in which the precursor gas stream, introduced vertically from the bottom and having a laminar flow, perpendicularly intersects the laser beam. Laser irradiation of the gaseous precursor is achieved by means of a focusing lens located in front of the reaction chamber. This technique is mainly employed for its advantages: (a) the well-defined interaction volume where no interactions with the reactor chamber walls occur, and hence, no contamination; (b) the production of very fine particles (usually less than 50 nm); (c) the small distribution of nanoparticle size; (d) control of growth rate and residence time in reaction zone; and (e) continuous working regime scalable to pilot station and even to industrial production.

As raw or auxiliary materials we employed volatile diethylzinc (ZnEt_2_, ≥52 wt % Zn content) and tetramethyltin (SnMe_4_, 95% purity) liquids from Sigma and sulfur hexafluoride (SF_6_), oxygen (O_2_), argon (Ar) (99.99999 vol %) and ethylene (C_2_H_4_) (99.999 vol %) bottled gases from Linde.

The reactive precursors used to obtain zinc-doped tin nanopowders, SnMe_4_ and ZnEt_2_, have no infrared absorption bands around the CO_2_ laser wavelength, which makes the introduction of a laser energy absorber (sensitizer) such as SF_6_ necessary. The SF_6_ molecules absorb part of the infrared laser beam energy and distribute it through collisions to the other gas species in the irradiation area. Thus, the reactive mixture very quickly reaches the temperature where the precursors start to decompose, forming Zn/F-doped Sn-based (and sometimes even SnF_2_) clusters. At the same time, due to the presence of an oxidative environment, the freshly formed clusters oxidize to SnO or SnO_2_ nanodomains. Also, a reference sample doped only with fluorine (noted FTO_st_) was synthesized in the absence of the zinc precursor, whereas the other reference sample (noted SnO_2_) was obtained also in the absence of Zn(C_2_H_5_)_2_ vapors, yet using C_2_H_4_ as a sensitizer instead of SF_6_ (see Table 4) to avoid both Zn and F doping.

**Table 4:** Experimental parameters for the different production conditions discussed in this article.

Sample	Central nozzle									Middle nozzle		External nozzle
	D_SF6_ [sccm]	D_Ar/ZnEt2_ [sccm]	D_ZnEt2_ [sccm]	D_Ar_ by pass Zn [sccm]	D_C2H4_ [sccm]	D_Ar/SnMe4_ [sccm]	D_C2H4/SnMe4_ [sccm]	D_SnMe4_ [sccm]	*R* = D_SnMe4/_D_ZnEt2_	D_O2_ [sccm]	D_Ar_int_ [sccm]	D_Ar_ext_ [sccm]

SnO_2_	–	–	–	–	5.25	–	5	2.50	–	15	50	1000
ZTO_st_	0.50	–	–	5.25	–	5	–	2.50	–	15	50	1000
ZTO_0.05_	0.50	1	0.05	4.20	–	5	–	2.50	50	15	50	1000
ZTO_0.15_	0.50	3.15	0.15	–	–	6.30	–	3.15	20	15	50	1000
ZTO_0.25_	0.50	5	0.25	–	–	5	–	2.50	10	15	50	1000
ZTO_0.44_	0.50	8.95	0.44	–	–	2.24	–	1.12	3	15	50	1000

In this study an inlet gas system of three concentric tubes was used. Different Ar flows served as carriers for the SnMe_4_ and ZnEt_2_ vapors. The main reactive stream consisting of SnMe_4_/ZnEt_2_ vapors and SF_6_ emerges into the reactor through the central nozzle tube. This central stream is directed towards the irradiated zone where the laser beam was focused to 1.5 mm diameter. An O_2_/Ar gas mixture passes through the middle annular gas admission, surrounding the central reactive stream in the irradiated area and creating a more-or-less oxidative environment. Simultaneously, the laminar flows of these two reactive streams are adjusted by an inert gas stream (Ar) that flows through the external (also annular) gas admission.

The total flow values for all three streams were adjusted in order to generate the same gas velocity above the admission nozzle (≈75 cm/s). In accordance with this restriction, the total central flow is maintained at 13.25 sccm, the median flow at 65 sccm and the external flow at 1000 sccm. Complementarily, two Ar flows are used to flush the ZnSe windows in order to impede the solid particle deposition on them. The reaction products that emerge from the reaction zone under the form of very fine, solid suspended particles were then entrained by the gas flow. The powder is trapped in the filter-containing collector and the gases, passing through the filter, leave the system with the aid of a vacuum pump.

The experiments take place at a constant laser power at 110 W and pressure of 450 mbar. Table 4 lists the main experimental parameters for which the change of the reactive and the dilution gas flows maintain a constant total inlet flow through the three zones at the entrance to the reaction chamber. In order to control the Zn doping level we have the possibility to divide the Ar flow designated to carry the ZnEt_2_ vapors; part of this flow can pass through the Zn-precursor-containing bubbler.

The powders resulting from the laser pyrolysis experiments were first characterized by XRD structural analysis (determination of crystalline phases and their mean dimension). Thus, X-ray measurements of Sn-based powders were performed at room temperature using the X-ray diffraction equipment X-Pert PRO MPD from PANalytical. The data is transmitted and then processed by the PANalytical X'Pert High-Score Plus software package. The XRD analytical method use the setup for polycrystalline powders in which the sample is exposed in front of a monochromatic X-ray beam having a variable incidence θ angle and a constant wavelength corresponding to the Kα copper line (λ = 1.5418 Å). Transmission electron microscopy (TEM and HRTEM) using a Tecnai F30 G2 (300 kV) instrument, was used to investigate the particle morphology, as well as the crystalline domains by selected area electron diffraction (SAED) analysis. Energy dispersion X-ray spectroscopy (EDX) was performed using a scanning electron microscopy (SEM-Inspect TM S50) with an acceleration voltage of 15 kV, using a SiLi detector cooled with liquid nitrogen. This method can give information on the elemental analysis (excluding hydrogen) on the samples.

The analysis of absorbance (*A*) data obtained by UV–vis–NIR spectrophotometry was performed on 0.01 g nanopowder sample suspended in 20 mL ethanol. The absorption spectra were registered using the computer-aided double-beam UV–vis–NIR spectrophotometer (Lambda 950, Perkin Elmer, USA) between 300–800 nm. The experimental spectral resolution was 0.05 nm for the UV–vis spectral domain. A measurement uncertainty of ±0.004% specified by the manufacturer for the device was considered.

The XPS measurements were performed in an ESCALAB Xi+ (Thermo SCIENTIFIC Surface Analysis) setup equipped with a multichannel hemispherical electron analyzer (dual X-ray source) operating with Al Kα radiation (*h*ν = 1486.2 eV), using C1s (284.4 eV) as the energy reference. XPS data were recorded by slightly pressing the powders on Si wafers. Then the samples were outgassed in the device prechamber at room temperature up to a pressure of <2 × 10^−8^ Torr in order to remove chemisorbed molecules from their surfaces. The surface chemical compositions and the oxidation states were estimated from the XPS spectra by calculating the integral of each peak after subtraction of the “S-shaped” Shirley-type background using the appropriate experimental sensitivity factors by means of “Avantage” software (version 5.978).

The acquisition of Raman spectra was achieved with a JASCO NRS-7200 Raman spectrometer using a blue laser source emitting at 532 nm.

In order to evaluate the powder resistivity, pellets with 10 mm metallized base diameter and 0.5 mm thickness were prepared by pressing 900 mg from each nanopowder up to 150 atm at room temperature. The resulting pellets have around 72% from SnO_2_ theoretical density.
